# Abducens nerve palsy with associated retinal involvement secondary to rickettsia typhi infection

**DOI:** 10.1186/s12348-021-00239-1

**Published:** 2021-03-22

**Authors:** Kaies Abderrahim, Sourour Zina, Molka Khairallah, Hager Ben Amor, Sana Khochtali, Moncef Khairallah

**Affiliations:** 1 Department of Ophthalmology, Medenine University Hospital, Faculty of Medicine, University of Sfax, Sfax, Tunisia; 2 Department of Ophthalmology, Fattouma Bourguiba University Hospital, Faculty of Medicine, University of Monastir, 5019 Monastir, Tunisia

**Keywords:** Rickettsiosis, Murine typhus, Fever, Abducens nerve palsy, Retinitis, SD OCT, Case report

## Abstract

**Objective:**

To report a case of abducens nerve palsy with associated retinal involvement due to rickettsia typhi infection.

**Material and methods:**

A single case report documented with multimodal imaging.

**Results:**

A 18-year-old woman with a history of high-grade fever was initially diagnosed with typhoid fever and treated with fluoroquinolone. She presented with a 5-day history of diplopia and headaches. Her best-corrected visual acuity was 20/20 in both eyes. Ocular motility examination showed left lateral gaze restriction. Lancaster test confirmed the presence of left abducens palsy. Fundus examination showed optic disc swelling in both eyes associated with superotemporal retinal hemorrhage and a small retinal infiltrate with retinal hemorrhage in the nasal periphery in the left eye. Magnetic resonance imaging (MRI) of the brain and orbits showed no abnormalities. A diagnosis of rickettsial disease was suspected and the serologic test for Richettsia Typhi was positive. The patient was treated with doxycycline (100 mg every 12 h) for 15 days with complete recovery of the left lateral rectus motility and resolution of optic disc swelling, retinal hemorrhages, and retinal infiltrate.

**Conclusion:**

Rickettsial disease should be considered in the differential diagnosis of abducens nerve palsy in any patient with unexplained fever from endemic area. Fundus examination may help establish an early diagnosis and to start an appropriate rickettsial treatment.

## Introduction

Rickettsial infections, are an important, but often underrecognized, cause of undifferentiated febrile illness [1]. Rickettsial agents are classified into three major groups: the spotted fever group, the typhus group, and the scrub typhus group. Murine typhus (MT), also called endemic typhus, is a febrile disease caused by Rickettsia typhi that is often misdiagnosed due to its non-specific presentation [1]. Patients with MT generally present with sudden onset of symptoms including acute sustained fever, severe headache, skin rash, chills, malaise, myalgia and anorexia [1]. Superficial retinitis, retinal vasculitis, and optic nerve involvement are the most common ocular manifestations of rickettsial disease [2, 3]. Ocular nerve palsies including the three and the six nerves have been rarely described [4–9]. We herein report a case of abducens nerve palsy associated with retinal involvement caused by murine typhus, which was initially misdiagnosed as typhoid fever.

## Case report

**Fig. 1:**
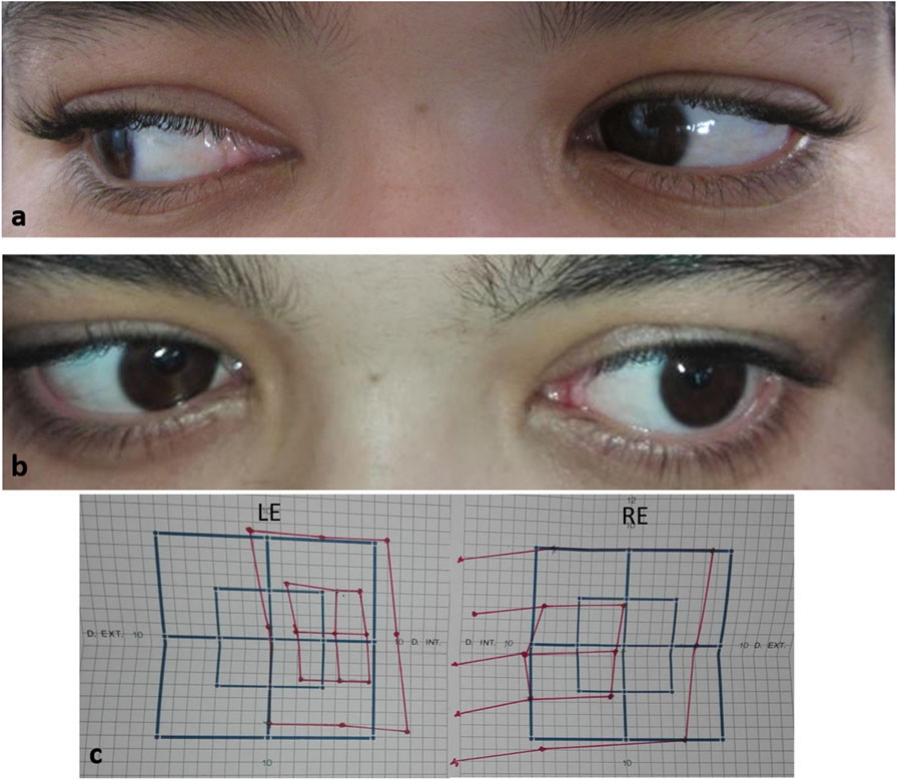
**a**, **b** Photography of the ocular motility examination reveals a left lateral gaze restriction (**b**)**.** Lancaster test confirms the presence of left abducens palsy (**c**)

**Fig. 2:**
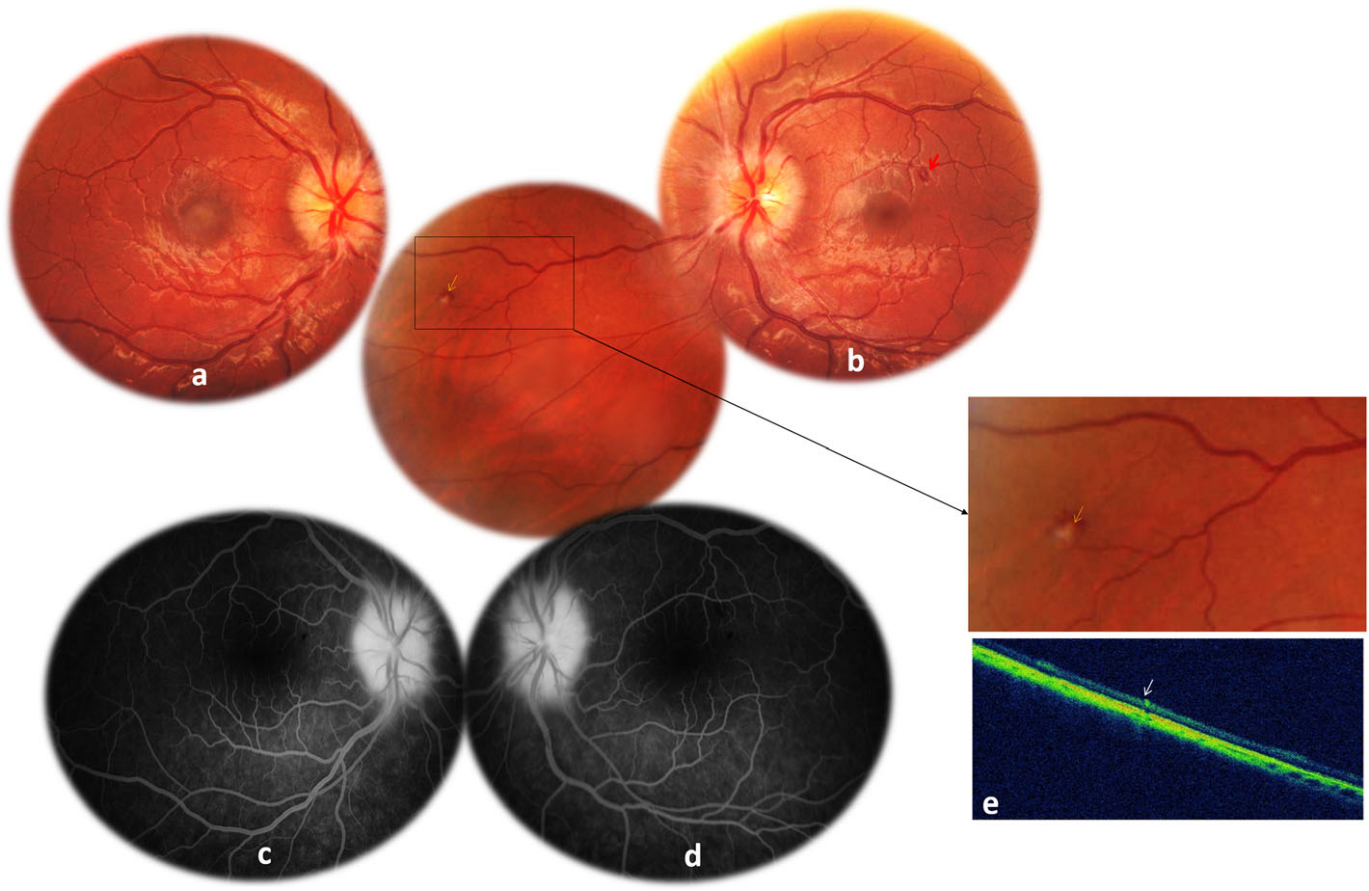
(**a**, **b**) Fundus photography at initial presentation showing optic disc swelling in both eyes. Note the presence of a superotemporal retinal hemorrhage (red arrow) and a small retinal infiltrate with retinal hemorrhage in the nasal periphery in the left eye (yellow arrow) more visible in the magnified rectangle (**b**). **c**, **d** Late-phase fundus fluorescein angiograms showing diffuse optic disc leakage in both eyes. **e** SD-OCT scan across the retinal infiltrate in the LE showing a focal area of inner retinal thickening and increased reflectivity with posterior shadowing (white arrow)

A 18-year-old woman presented to our department with a 5-day history of diplopia and headaches. She had developed 15 days earlier a high-grade fever, and had been diagnosed as having typhoid fever and treated with fluoroquinolone. On examination, the patient’s best-corrected visual acuity was 20/20 in both eyes. Ocular motility examination revealed a left lateral gaze restriction (Fig. 1 a and b). Lancaster test confirmed the presence of left abducens palsy (Fig. 1 c). There was no relative afferent pupillary defect. The anterior chamber and the vitreous were quiet in both eyes. Intraocular pressure was normal bilaterally. Fundus examination showed optic disc swelling in both eyes (Fig. 2 a and b). There also were a superotemporal retinal hemorrhage and a small retinal infiltrate with retinal hemorrhage in the nasal periphery in the left eye (Fig. 2 b). Late-phase fundus fluorescein angiograms showed diffuse optic disc leakage in both eyes (Fig. 2 c and d). Macular SD-OCT was unremarkable in both eyes. SD-OCT scan across the retinal infiltrate in the LE showed a focal area of inner retinal thickening and increased reflectivity with posterior shadowing **(**Fig. 2 e).

**Fig. 3:**
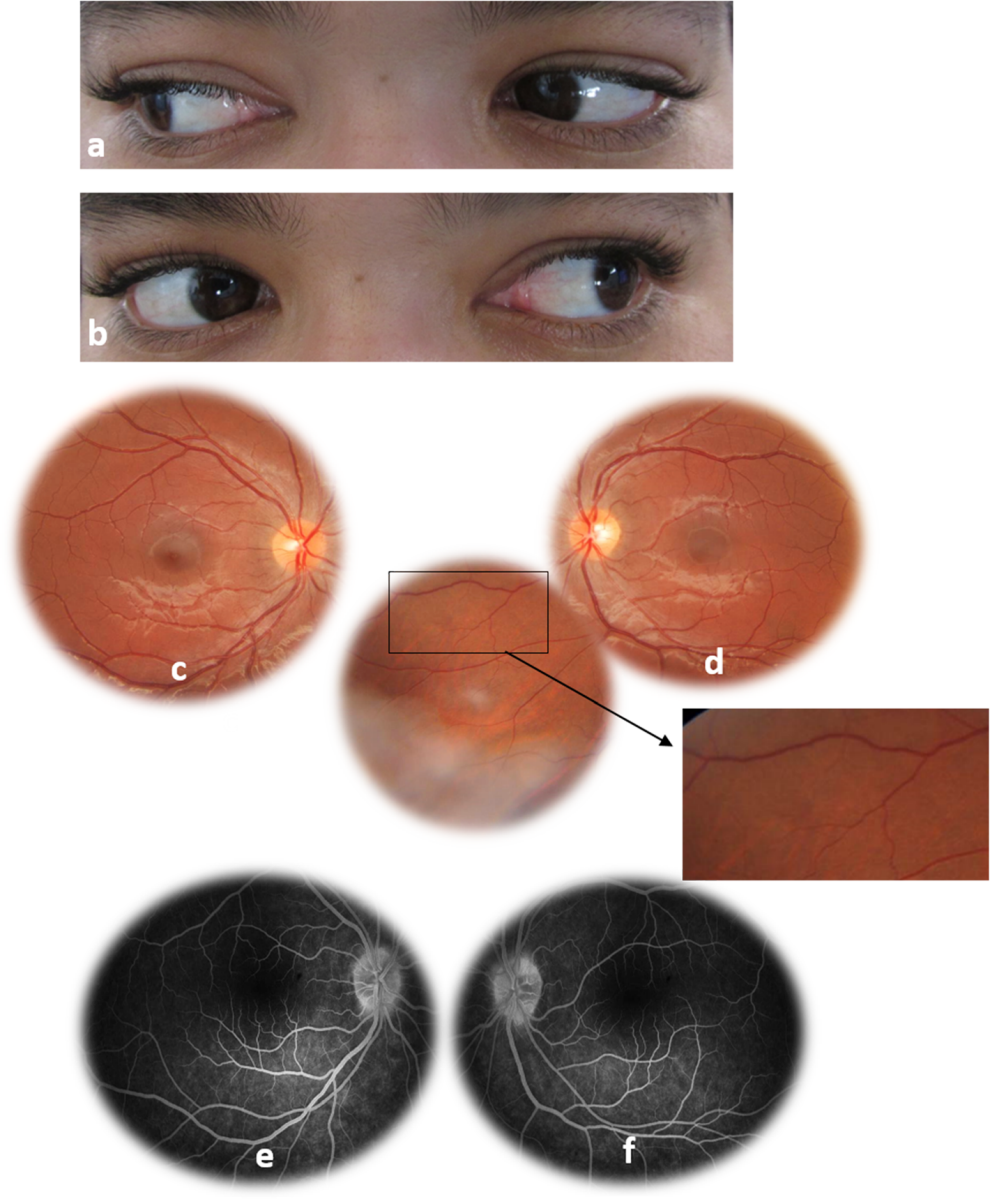
**a**, **b** Photography of the ocular motility examination, two weeks after initiation of doxycycline therapy, reveals recovery of the left lateral rectus motility (**b**)**. c**, **d** Fundus photography showing the complete resolution of optic dis swelling, retinal hemorrhages, and retinal infiltrate. **e**, **f** Late-phase fundus fluorescein angiography showing the resolution of optic disc leakage in both eyes

Results of physical examination, including neurologic evaluation, were unremarkable. Laboratory work-up showed raised C-reactive protein (CRP), mild thrombocytopenia, and mildly elevated liver enzymes. Magnetic resonance imaging (MRI) of the brain and orbits showed no abnormalities. Rickettsial disease was suspected, and the serologic test for Richettsia Typhi was positive. The patient was treated with doxycycline (100 mg every 12 h) for 15 days. Symptoms rapidly improved, with complete resolution of diplopia and headaches. Two weeks after initiation of doxycycline therapy, ocular motility examination revealed recovery of the left lateral rectus motility **(**Fig. 3 a and b). Fundus examination showed the complete resolution of optic disc swelling, retinal hemorrhages, and retinal infiltrate **(**Fig. 3 c and d). Late-phase fundus fluorescein angiography showed the resolution of optic disc leakage in both eyes **(**Fig. 3 e and f).

## Discussion

In this report, we describe a case of abducens nerve palsy associated with rickettsial infection. The patient initially presented with high-grade fever and was initially diagnosed as having typhoid fever and treated accordingly. The diagnosis of rickettsial infection was initially missed in the absence of typical skin rash. Fundus examination, showing fairly typical superficial retinal infiltrate, led us to suspect rickettsial disease**.** The diagnosis was confirmed by the positivity of the serologic test for Richettsia Typhi. The patient received a 15-day course of doxycycline therapy, with subsequent favorable response.

MT is frequently misdiagnosed, being a major cause of fever of unknown origin in numerous geographic regions. The initial clinical presentation of MT develops after an 8- to 16-day incubation period, and includes non-specific clinical manifestations, such as high fever, headaches and an often poorly visible maculopapular rash [1, 2]. However, this classic triad was found in only one-third of infected patients [1]. Furthermore, the contact with vectors were rarely mentioned by infected patients and flea bites are occasionally found on physical examination [1]. The clinical course of MT is generally benign and self-limited [1, 2]. Complications are uncommon including pneumonia, renal insufficiency and neurologic involvement [1, 10].

Besides superficial retinitis, an array of other ocular manifestations has been described in association with rickettsial infection including retinal vasculitis, anterior uveitis, optic disc swelling, optic neuropathy, and Parinaud ocular glandular syndrome [2, 3]. Neurologic complications occur in 2-10% of patients with MT, and they include aseptic meningitis, meningoencephalitis and rarely, cranial nerve palsy [1, 10]. There are only a few reported cases of ocular nerve palsy associated with rickettsial disease, mainly involving the third and the sixth nerves [4–9]. Abducens nerve palsy was associated with meningoencephalitis and/or raised intracranial pressure in the majority of reported cases (Table 1) [5–7]. However, isolated abducens nerve palsy without obvious meningoencephalitis was reported only twice (Table 1) [8, 9]. This could result from microvascular infarction involving the cranial nerve, reflecting the marked tropism of rickettsial organisms for the endothelial cells of small vessels. The latter mechanism could explain the development of abducens nerve palsy in our patient, but a raised intracranial pressure could not be excluded. Therefore, bilateral optic disc swelling in our patient might correspond to papillitis or papilledema.

**Table 1:** Reported cases of murine typhus related abducens nerve palsy

Author	Country	Age (years)	Gender	Fever	Headache	Skin rash	Duration of symptoms before onset of ocular nerve palsy	Abducens nerve palsy laterality	Associated complications
Simon NG, et al. [5]	Australia	20	Male	+	+	–	10	Bilateral	Seizure, Meningoencephalitis
Masalha R, et al. [6]	Israel	22	Female	+	+	–	14	Right	Mild subacute meningoencephalitis
Hsu CH, et al. [8]	Indonesia	31	Male	+	+	–	2	Right	Liver dysfunction
Moy WL, et al. [7]	Singapore	39	Male	+	–	–	9	Right	Liver dysfunction, Mild meningitis
Moy WL, et al. [7]	Indonesia	27	Female	+	+	–	4	Bilateral	Meningoencephalitis and ventilator associated pneumonia
Lin Ting-Yang, et al. [9]	Taiwan	39	Female	+	+	+	9	Left	Liver dysfunction
Present case	Tunisia	18	Female	+	+	–	10	Left	Liver dysfunction

MT should be considered in the differential diagnosis of abducens nerve palsy in any patient with unexplained fever living in or travelling back from a specific endemic area. Ophthalmic examination, showing typical fundus changes, may be helpful to establish an early diagnosis and to start an appropriate rickettsial treatment.

## Data Availability

The datasets used and/or analysed during the current study are available from the corresponding author on request.

## Abbreviations

CRP: C-reactive protein**,**
MRI: Magnetic resonance imaging
MT: Murine typhus
